# Genome Sequence of *Pseudomonas azelaica* HBP1, Which Catabolizes 2-Hydroxybiphenyl Fungicide

**DOI:** 10.1128/genomeA.01248-13

**Published:** 2014-02-13

**Authors:** José L. García, Daniel Rozas, Carlos del Cerro, Juan Nogales, Magdy El-Said Mohamed, Eduardo Díaz

**Affiliations:** a Environmental Biology, Centro de Investigaciones Biológicas, Consejo Superior de Investigaciones Científicas, Madrid, Spain; b R&DC, Saudi Aramco, Dhahran, Saudi Arabia

## Abstract

*Pseudomonas azelaica* HBP1 (DSM 8897) is one of the few bacteria able to completely mineralize the 2-hydroxybiphenyl biocide. Here, we report the draft genome sequence of this strain (7.4 Mbp; G+C content, 63.5%) and the findings obtained from its genome annotation.

## GENOME ANNOUNCEMENT

2-Hydroxybiphenyl (2-HBP) is a bulk chemical with biocidal properties; it is primarily used as an agricultural fungicide, but it is also used in hospital and household disinfectants and in personal health care products. In addition, 2-HBP is the main by-product of the biodesulfurization of oil, produced through microbial conversion of dibenzothiophene (1). Due to its extensive use, 2-HBP can be found in persistent low quantities in sewage effluents (2). The bacterial degradation of 2-HBP is rare, and one of the few bacteria known to completely metabolize 2-HBP is *Pseudomonas azelaica* HBP1 (DSM 8897), which was isolated from a wastewater treatment plant (3).

The genome of *P. azelaica* HBP1 (DSM 8897) has been sequenced using the 318 Chip and 400-bp chemistry Ion Torrent PGM platform, as per the manufacturer’s instructions. The preliminary assembly of raw reads was performed using Newbler software from Roche. This assembly was manually revised and improved, obtaining a quality draft of 212 contigs. The genome was structurally and functionally annotated using RAST (4), an automated genome annotation system, and the functions, names, and general properties of the gene products were predicted using this method. *P. azelaica* HBP1 has one of the largest *Pseudomonas* genomes (7.4 Mbp in size) and contains 63.5% G+C content, 48 RNAs, and 7,388 coding sequences. The HBP1 strain harbors at least one megaplasmid.

The capacity of *P. azelaica* HBP1 to metabolize 2-HBP (and 2,2′-dihydroxybiphenyl) to benzoate (salicylate) and 2-hydroxy-pentadienoate correlates with the expression of the *hbp* gene cluster (5). Remarkably, the analysis of the complete genome revealed that the *hbp* cluster is located within a 177-kbp-long integrative conjugative element (ICEhbp), whose G+C content (60.2%) suggests an evolutionary origin different from that of the rest of the chromosome. The ICEhbp also contains the genes for the *meta*-cleavage pathway of salicylate, the metabolism of 2-hydroxy-pentadienoate, and (methoxy)phenylpropanoid catabolism via the β-ketoadipate *ortho*-cleavage pathway. At least two different *ben-cat* clusters for benzoate degradation via the β-ketoadipate *ortho*-cleavage pathway can be identified outside the ICEhbp element.

JSpecies (6) comparison of *P. azelaica* HBP1 and the closely related strains *Pseudomonas denitrificans* ATCC 13867 (GenBank accession no. NC_020829) and *Pseudomonas nitroreducens* TX1 (accession no. AMZB00000000) gives average nucleotide identity based on BLAST (ANIb) values of 87.45% and 90.25%, respectively (ANIb aligned, 56.27% and 70.45%), and ANI based on MUMmer (ANIm) values of 89.53% and 91.37%, respectively (ANIm aligned, 57.26% and 73.99%).

### Nucleotide sequence accession number.

The *P. azelaica* HBP1 (DSM 8897) genome sequence has been submitted to GenBank under the accession no. AZRU00000000.
